# Coping After Breast Cancer: Protocol for a Randomized Controlled Trial of Stress Management eHealth Interventions

**DOI:** 10.2196/47195

**Published:** 2023-05-24

**Authors:** Karianne Svendsen, Lise Solberg Nes, Anders Meland, Ine Marie Larsson, Ylva M Gjelsvik, Elin Børøsund, Christine M Rygg, Tor Åge Myklebust, Kristin V Reinertsen, Cecilie E Kiserud, Helle Skjerven, Michael H Antoni, Trudie Chalder, Ingvil Mjaaland, Linda E Carlson, Hege R Eriksen, Giske Ursin

**Affiliations:** 1 Cancer Registry of Norway Oslo University Hospital Oslo Norway; 2 Department of Nutrition Institute of Basic Medical Sciences University of Oslo Oslo Norway; 3 Lipid Clinic Oslo University Hospital Oslo Norway; 4 Department of Digital Health Research Division of Medicine Oslo University Hospital Oslo Norway; 5 Institute of Clinical Medicine Faculty of Medicine University of Oslo Oslo Norway; 6 Department of Psychiatry and Psychology College of Medicine and Science Mayo Clinic Rochester, MN United States; 7 Department of Sport and Social Sciences School of Sport Sciences Oslo Norway; 8 Department of Nursing and Health Sciences Faculty of Health and Social Sciences University of South-Eastern Norway Drammen Norway; 9 National Advisory Unit for Late Effects After Cancer Department of Oncology Oslo University Hospital Oslo Norway; 10 Section for Breast and Endocrine Surgery Department Vestre Viken Hospital Trust Drammen Norway; 11 Department of Psychology Sylvester Comprehensive Cancer Center University of Miami Miami, FL United States; 12 Department of Psychological Medicine King's College London London United Kingdom; 13 Department of Oncology and Hematology Stavanger University Hospital Stavanger Norway; 14 Department of Oncology University of Calgary Calgary, AB Canada; 15 Department of Psychology University of Calgary Calgary, AB Canada; 16 Department of Sport, Food and Natural Sciences Western Norway University of Applied Sciences Bergen Norway; 17 Department of Preventive Medicine Keck School of Medicine University of Southern California Los Angeles, CA United States

**Keywords:** breast cancer, stress management, mindfulness, cognitive behavioral therapy, psychosocial, StressProffen, RCT, eHealth, digital, intervention

## Abstract

**Background:**

One-third or more of breast cancer survivors report stress and other psychological and physical complaints that can negatively impact their quality of life. Psychosocial stress management interventions, shown to mitigate the negative impact of these complaints, can now be delivered as accessible and convenient (for the patient and provider) eHealth interventions. In this randomized controlled trial (RCT), Coping After Breast Cancer (CABC), 2 modified versions of the stress management eHealth intervention program StressProffen were created: one with predominantly cognitive behavioral stress management content (StressProffen-cognitive behavioral therapy intervention [StressProffen-CBI]) and another with predominantly mindfulness-based stress management content (StressProffen-mindfulness-based intervention [StressProffen-MBI]).

**Objective:**

This study aims to investigate the effects in breast cancer survivors of using StressProffen-CBI and StressProffen-MBI compared with a control group (treatment as usual).

**Methods:**

Women diagnosed with breast cancer (stage I-III, unequivocally human epidermal growth factor receptor 2–positive or estrogen receptor-negative tumors) or ductal carcinoma in situ (DCIS) aged 21-69 years who completed the Cancer Registry of Norway–initiated health survey on quality of life are invited to the CABC trial about 7 months after diagnosis. Women who give consent to participate are randomized (1:1:1) to either the StressProffen-CBI, StressProffen-MBI, or control group. Both StressProffen interventions consist of 10 modules of stress management content delivered through text, sound, video, and images. The primary outcome is between-group changes in perceived stress at 6 months, assessed with Cohen 10-item Perceived Stress Scale. The secondary outcomes comprise changes in quality of life, anxiety, depression, fatigue, sleep, neuropathy, coping, mindfulness, and work-related outcomes approximately 1, 2, and 3 years after diagnosis. Long-term effects of the interventions on work participation, comorbidities, relapse or new cancers, and mortality will be assessed using data from national health registries.

**Results:**

Recruitment is scheduled from January 2021 to May 2023. The goal is to recruit 430 participants (100 in each group). As of April 14 2023, 428 participants have been enrolled.

**Conclusions:**

The CABC trial is possibly the largest ongoing psychosocial eHealth RCT in patients with breast cancer. If 1 or both interventions prove to be effective in reducing stress and improving psychosocial and physical complains, the StressProffen eHealth interventions could be beneficial, inexpensive, and easily implementable tools for breast cancer survivors when coping with late effects after cancer and cancer treatments.

**Trial Registration:**

Clinicaltrials.gov NCT04480203; https://clinicaltrials.gov/ct2/show/NCT04480203

**International Registered Report Identifier (IRRID):**

DERR1-10.2196/47195

## Introduction

### Breast Cancer Treatment and Long-Term Complaints

Breast cancer survival rates largely depend on cancer subtype, stage, treatment, and age. The 5-year relative survival rate for individuals with early breast cancer is firmly increasing and has exceeded 90% in most Western countries due to advances in diagnostics and treatment [1,2]. In Norway, nationwide mammography screening programs combined with high breast cancer awareness have contributed to most patients being diagnosed with stage I with 100% 5-year survival rate [2].

Supportive therapies have improved and thus reduced the immediate side effects of adjuvant systemic therapies. Nonetheless, breast cancer diagnosis and treatment may still cause substantial psychosocial complaints (stress, distress, anxiety and depression), pain, fatigue [3,4], and several physical complaints that usually emerge during treatment and often persist over a longer term [5,6].

These complaints can affect the ability to manage thoughts, feelings, and behavior [7,8] and negatively impact the individual’s perceived physical, mental, and social well-being, often assessed through measures of health-related quality of life [9-11]. A breast cancer diagnosis and therapy may also have persistent negative effects on work and increase the risk of both short-term sick leave [12-14] and long-term disability pension [15-24], thus resulting in a lower workforce participation years following the diagnosis [25]. With an increasing prevalence of cancer worldwide [26], interventions to improve quality of life for cancer survivors are therefore important [27].

### Psychosocial Stress Management Interventions

Psychosocial stress management interventions have been shown to mitigate short- and long-term complaints following a cancer diagnosis and treatment [9,28,29]. This includes improvement of negative psychosocial impacts of breast cancer [30-32], reduction of stress and stress-related symptoms [31,33,34], anxiety [31], depression [10,31,35], fatigue [31,35-37], insomnia [37-39], and pain [34], as well as improvement in quality of life [9,37,40,41]. The prevalence of distress and the potential negative impact of unrecognized and untreated distress in patients with cancer are widely recognized [42], and so is the potential effectiveness of psychosocial interventions for stress and distress management [43].

Psychosocial stress management interventions typically include components of cognitive behavioral therapy (CBT) and mindfulness techniques, designed to teach patients strategies for managing stress [27,44]. The basis of CBT is understanding that cognitions mediate the relationship between life events and emotional reactions [45]. CBT strategies target unhelpful or maladaptive cognitions with various techniques and behavioral strategies to moderate emotional responses and unhelpful coping behavior [45]. CBT-based interventions (CBIs) often combine aspects such as cognitive restructuring, coping effectiveness training, assertiveness, and anger management skills with relaxation (eg, muscle relaxation and imagery) [46].

Mindfulness-based interventions (MBIs) aim to cultivate present-moment awareness coupled with attitudes of acceptance, nonjudging, and nonattachment, through both formal (eg, awareness of breathing, sitting, walking, body scan) and informal (eg, mindfulness in everyday life) mindfulness practices. MBIs are typically adapted from the Mindfulness-Based Stress Reduction program of Kabat-Zinn [31,47]. One such adaptation, mindfulness-based cancer recovery [29], is specifically designed for patients and survivors of cancer [48].

Although often used in combination, there are distinct theoretical and methodological differences between CBIs and MBIs for stress management. Although one of the core components in CBIs is to help modify “unhelpful” ways of thinking, MBIs fall within a group of “acceptance-based therapies” and focus on adopting a state of nonjudgmental awareness and acceptance of the present moment, without a specific intent to modify thoughts or behavior [49]. CBIs such as cognitive behavioral stress management interventions have been used extensively in studies with breast cancer survivors [9,10] with positive effects in both short and long term [9,10]. This includes a reduction in psychosocial and physical complaints and improvement of physical and mental quality of life [9], up to 15 years after the intervention [50]. MBIs have shown positive short-term effects on psychosocial and physical complaints and improvement in measures of quality of life [51-54], but the long-term effects are either inconclusive or have not been studied [37]. Furthermore, whether the effects of CBIs and MBIs on stress management are comparable is unknown, as no known direct comparison between the intervention types has been conducted.

The optimal window for when to deliver stress management interventions has not been established [55]. Many psychosocial interventions have been conducted short term following a breast cancer diagnosis as this represents an acute period of stress that may influence treatment response and relevant clinical outcomes. There have also been successful psychological interventions implemented after surgery, although not as frequently as after other types of treatment for breast cancer survivors [55].

### e-Health Interventions

Although in-person face-to-face delivery of psychosocial stress management interventions has been the most common, technical innovations that have accelerated due to the COVID-19 pandemic have resulted in increased use of eHealth interventions in clinical practice [55]. There are advantages to developing eHealth versions of established stress management interventions that extend beyond an adaptation to the restrictions imposed by the pandemic. eHealth interventions are often less expensive to implement after the initial production costs, and are more accessible for people living in remote areas. These can also be more convenient for persons with movement restrictions or other reasons for not being able to see health care providers face-to-face [56]. Studies testing eHealth psychosocial stress management interventions have been considered acceptable and feasible for breast cancer survivors [56,57], with positive effects on quality of life in patients with cancer following diagnosis [58]. However, more research is needed [55], as the effects of psychosocial eHealth interventions, in general in cancer survivors, have been mixed and inconclusive [59-63]. Furthermore, there is a need for studies testing stress management eHealth interventions in larger samples, after the initial phase of medical treatment, and with longer follow-up than previous research [64].

The Coping After Breast Cancer (CABC) trial was designed to test the effect of 2 eHealth-based stress management interventions (CBI and MBI groups), in comparison to a treatment as usual (TAU) control condition, on improving psychosocial factors, physical complaints, and work-related outcomes in breast cancer survivors. With long-term follow-up on work participation, comorbidities, relapse or new cancers, and mortality, the trial addresses the overall aim of validating eHealth stress management interventions to facilitate future implementation.

## Methods

### Objective and Hypotheses

The overall objective of the CABC trial is to investigate the effects of using eHealth interventions with cognitive behavioral stress management content (CBI group) or mindfulness-based stress management content (MBI group) in breast cancer survivors compared with a TAU control group (TAU group).

### Specific Hypotheses

- The CBI and MBI groups will achieve a statistically significant larger reduction in perceived stress after 6 months of access to the interventions (13 months after diagnosis) compared with the TAU control group (*primary outcome*).
- The CBI and MBI groups will achieve a significantly larger reduction in perceived stress, distress, fatigue, peripheral neuropathy, vaginal symptoms, and arthralgia and a significantly larger improvement in quality of life, mindfulness, sleep, coping, and work-related outcomes 13, 24, 35, and 36 months after diagnosis compared with the TAU control group (*secondary outcomes*).
- Additionally, exploratory analyses will be conducted to investigate the following:
  characteristics of study responders and intervention completers versus nonresponders and noncompleters;
  predictors of intervention effects;
  potential differences in intervention effects between the CBI and MBI groups;
  long-term consequences of the interventions related to work participation; and
  long-term effects of the interventions on comorbidities, relapse or new cancers, and mortality.

### Study Design and Setting

The CABC trial is a randomized controlled trial (RCT; ClinicalTrials.gov identifier NCT04480203) assessing the effects of 2 stress management eHealth interventions compared with TAU in breast cancer survivors. The trial is conducted at the Cancer Registry of Norway (CRN) at Oslo University Hospital. The eHealth interventions are delivered through the mobile app StressProffen [65], which was developed, and is managed, by a team led by 2 of the coauthors (LSN and EB) at the Department of Digital Health Research at Oslo University Hospital.

**Figure 1:**
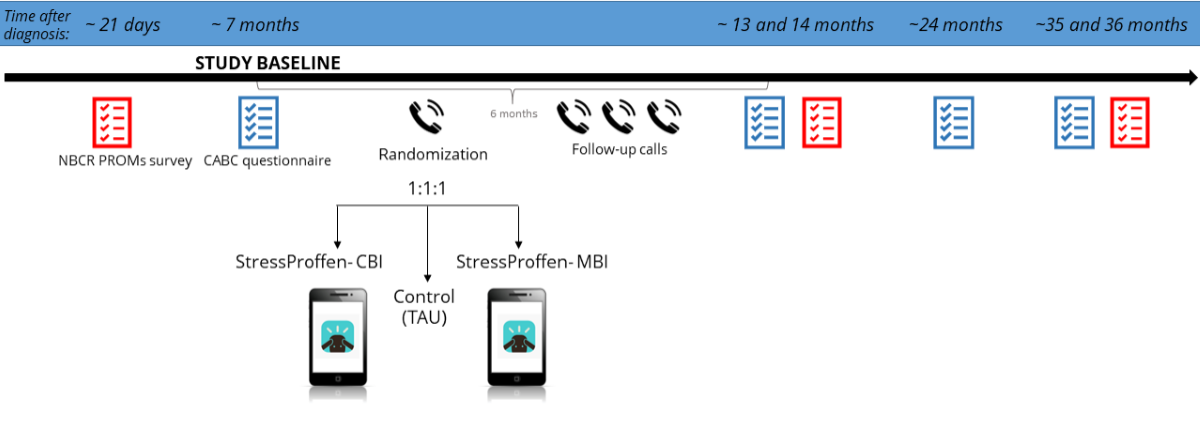
Overview of milestones in the CABC RCT. Study baseline is approximately 7 months after breast cancer diagnosis. Eligible participants who have completed the CRN PROMs survey, consented to the CABC study, and completed baseline questionnaires are randomized to either the StressProffen-CBI, StressProffen-MBI or a control group (TAU). Recruitment is scheduled from January 2020 to May 2023. Pictures from colourbox.com and StressProffen. CRN PROMs survey: The Cancer Registry of Norway Patient Reported Outcome Measures; CABC: Coping After Breast Cancer; CBI: cognitive behavioral intervention; MBI: mindfulness-based intervention; NBCR: Norwegian Breast Cancer Registry; RCT: randomized controlled trial; TAU: treatment as usual.

The study protocol of the CABC trial is presented according to the SPIRIT (Standard Protocol Items: Recommendations for Interventional Trials) initiative [66] (Multimedia Appendix 1). The study outline is shown in Figure 1 and described in further detail in the following sections.

### Participants and Eligibility

#### The Cancer Registry of Norway

Study participants with incident breast cancer are identified and included from the CRN. The CRN has recorded incident cancer cases in Norway since 1952 with accurate and almost complete ascertainment of cases (99% complete on a minimum of diagnostic information on solid tumors [2]). The CRN hosts the Norwegian Breast Cancer Registry that has detailed information on breast cancer diagnoses extracted from pathology reports, and details on treatment including the type of breast and axillary surgery, radiotherapy, and systemic therapy. Nonetheless, as of 2020, the CRN also collects and stores data on patient-reported outcome measures (PROMs). The CRN PROMs survey is an ongoing, digital survey sent through the official Norwegian health portal Helsenorge [67] or an official digital mailbox (Digipost/eBox) to patients with breast cancer and age-matched controls approximately 21 days, 14 months, and 36 months following diagnosis. Currently, 88% of eligible patients with breast cancer are invited to this survey, and the response rates were 52% in 2020 and 49% in 2021 [68]. Specific data from the CRN PROMs survey will be included as outcomes in this study, and the survey content is therefore described further below.

#### CABC Trial Eligibility

Women who are aged between 21 and 69 years when diagnosed with subtypes of invasive breast cancer, International Classification of Diseases, 10th revision (ICD-10) code C50, or ductal carcinoma in situ (DCIS), ICD-10 code D05.9, in the period from 2020 to 2023, and who have completed the initial CRN PROMs survey are eligible for inclusion in the CABC trial, at about 7 months (6-9 months) after diagnosis. The timing was chosen to ensure that most patients have completed the first part of their treatment, and that details about the breast cancer subtype have been coded in the CRN.

See Textbox 1 for an overview of the inclusion criteria. Participants in the intervention groups must also have access to, and be able to use, a smartphone or a tablet. There are no restrictions regarding types of treatment, nor any geographical restrictions, given the digital nature of the intervention.

The upper age limit of 69 years was chosen to correspond with the upper age limit of the participants in the Norwegian breast screening program. To avoid overlap with 2 ongoing clinical trials in Norwegian patients with breast cancer (EMIT, ClinicalTrials.gov Identifier: NCT03904173 and OPTIMA [no registration number]), recruitment of the invasive cases in the CABC trial is restricted to those with stage I-III estrogen receptor (ER)–negative or human epidermal growth factor receptor 2 (HER2)–positive tumors, or a combination of ER+ and HER2–. This is typically about 15% of all breast cancer cases (unpublished data from the CRN during 2020-2021).

**Inclusion and exclusion criteria for the Coping after Breast Cancer trial.**

**Inclusion criteria**

- Women;
- Incident: ductal carcinoma in situ or breast cancer stage I-III, unequivocally human epidermal growth factor receptor 2–positive or estrogen receptor–negative tumor(s);
- Age 21-69 years;
- Residing in Norway;
- Submitted the initial Cancer Registry of Norway patient-reported outcome measures (CRN PROMs) survey, which is sent 21 days after diagnosis. This also implies that they are fluent in Norwegian and are active users of the digital portal: Helsenorge [67] or have a digital mailbox.
- Submitted the initial Coping after Breast Cancer (CABC) survey, which is also sent 21 days after diagnosis. This also implies that they are fluent in Norwegian and are active users of the digital portal: Helsenorge or have a digital mailbox (that imply consent to participation).

#### CABC Trial Enrollment

Study recruitment is scheduled from January 2021 to May 2023 with patients diagnosed over the previous year. Participants are continually recruited at approximately 7 months after their breast cancer diagnosis.

**Figure 2:**
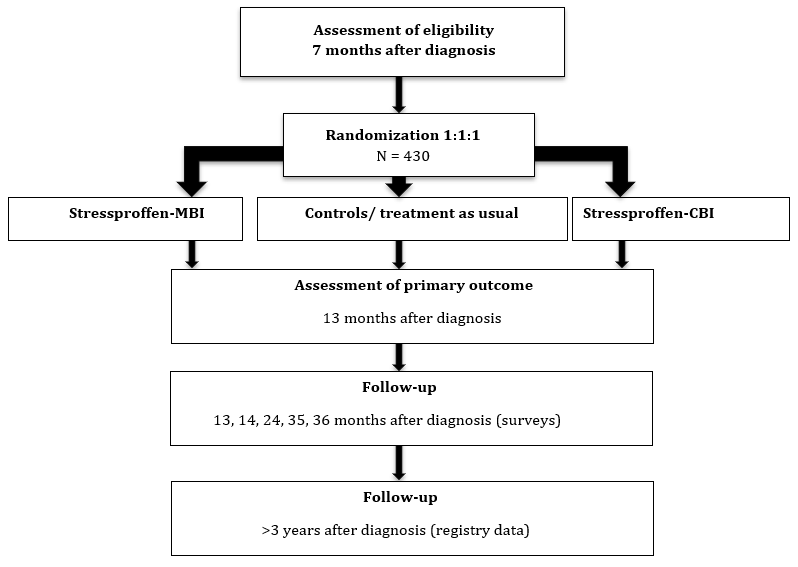
Flowchart of inclusion of study participants invited ≈7 months after diagnosis. CBI: cognitive behavioral intervention. MBI: mindfulness-based intervention.

Invitations are sent every week (except during holiday periods) along with the study-specific CABC survey. In total, 430 participants will be enrolled in the trial (Figure 2).

### Randomization

Once an eligible participant has submitted the CABC survey at baseline (that also implies consent to participate), the participant is allocated to 1 of the intervention groups or the control group in the ratio 1:1:1 (Figure 1). The randomization algorithm uses a variant of block randomization, with no stratification, where block sizes vary between 12, 18, 21, and 27. The output of the randomization algorithm for each participant is a string representing an access code to 1 of the 2 interventions (each intervention has a unique access code), or a string indicating that the participant was randomized to the control group. The access code is used to download the StressProffen program (an app). The randomization is performed in R studio version 1.2.1335 (The R Foundation for Statistical Computing).

Intervention identification is not possible from the access code, and participants in the 2 intervention groups are not informed to which of the intervention they have been assigned to. However, an informed participant may detect which group they are in by identifying content when using the assigned app. Hence the trial is single blinded.

After randomization, participants randomized to 1 of the intervention groups receive a letter containing information about the scheduled time for the first phone call. For participants randomized to the control group, the letter contains information about the timing of further follow-up surveys.

### StressProffen Interventions

The interventions used in this trial are 2 modified versions of an evidence-based stress management eHealth intervention program (StressProffen) developed for cancer survivors [65,69-71]. For privacy reasons, there is, however, no mention of the word “cancer” in either intervention.

The original StressProffen program includes both CBT and mindfulness. It contains psychoeducational materials (eg, about stress, coping, social support, anger management, assertiveness, and health behaviors) and a variety of exercises, including exercises related to how to cope with challenging thoughts, feelings, and bodily states (eg, positive self-talk, diaphragmatic breathing, progressive muscle relaxation, guided imagery, mindfulness, and meditation) [65].

For the CABC trial, the project team modified the StressProffen program and developed 2 distinct stress management interventions: a CBI (StressProffen-CBI) and an MBI (StressProffen-MBI) (E. Børøsund et al, unpublished data, 2022).

Both interventions contain 10 modules of stress management education material and exercises delivered through text, sound, video, and images/pictures [65]. After 1 module is completed, there is a 3-day delay before the next module is available. The purpose of this delay is to encourage the participant to spend time reflecting and practicing previous content and exercises. The program has several features that make it easy to use, such as the ability to mark exercises as favorites, offline mode, and the possibility to set reminders [65].

Modification of the content/content development was conducted by 4 subject matter experts (AM, HRE, LSN, and EB), an epidemiologist (GU), and a content editor (CMR). As in the original StressProffen intervention program, the StressProffen app was used for both interventions. General information (ie, text unrelated to CBI and MBI) was retained from the original StressProffen program and reused for both interventions, and the team strived to balance the number and length of the other modules and subsections, exercises, figures, and models between the 2 interventions.

The content of the final interventions relied on the original StressProffen program, with most of the original content being retained in the CBI version, but with some adjustments, including minor therapeutic language adjustments. More content adjustments and revisions were made for the MBI version, with new developed content from the project team experts, as well as inclusion of adapted content (ie, translation and modifications to fit the language and structure) from the Mindfulness-Based Cancer recovery manual [29]. The adaptation included translation and modifications to fit the language and structure used in the current intervention (E. Børøsund et al, unpublished data, 2022).

All participants in the intervention groups have access to the assigned StressProffen interventions for the entire study period and in the future for as long as Oslo University Hospital manages the app. The control group receives access to StressProffen upon request when their participation in the trial is finished.

### Potential Intervention Harms

The CBI and MBI interventions have no known harms. A feeling of disappointment might arise if the participant does not feel that their stress level has been reduced during the study period. Although the vast majority of literature says otherwise, it cannot be completely ruled out that meditation-based therapies may lead to distress [72].

As the participants do not have access to individual therapy through the study, participants in both intervention groups are encouraged to seek professional help from their primary caregiver, should they wish/feel a need to do so.

### Follow-up

The initial phone call to participants in the intervention groups is scheduled shortly after randomization and consists of a structured introduction to the StressProffen program lasting 15-20 minutes (Figure 1). During this phone call, participants download the StressProffen app, obtain, and enter their access code, and consequently receive access to download 1 of the 2 interventions. Participants are encouraged to start using the app immediately after the phone call.

After the initial phone call, there are 3 additional follow-up phone calls (approximately 1, 3, and 6 weeks following the initial phone call) to increase adherence to the protocol, especially to support motivation and prevent dropouts by checking how participants are doing with the intervention and resolving any technical issues. For the follow-up phone calls, 3 weekly attempts to reach the participants are undertaken. During the final phone call at 6 weeks, participants are informed that further follow-up in the CABC trial will consist of annual surveys over the next 3 years (Figure 1).

### Communication

In addition to the scheduled follow-up calls, participants are encouraged to call the study telephone number with any questions they may have. The cancer advisory unit at the patient advocate organization, the Norwegian Cancer Society [73], and the Breast Cancer Oncology Group (Norwegian Breast Cancer Group) [74] also have basic information about the CABC trial, in case the participants call them for support. Furthermore, the CRN and the Norwegian Breast Cancer Society share information about the trial occasionally through their social media platforms and newsletters. These channels will also be used to inform people about published results. In addition to scientific publications and presentations of results at scientific conferences, results will be communicated to the public through various media outlets after the study end. Any changes to the protocol will be recorded simultaneously at the ClinicalTrials.gov registry [75].

### Outcome Measures

#### Surveys

Outcomes are assessed with 2 digital surveys: the ongoing CRN PROMs survey and the study-specific CABC survey (Table 1).

**Table 1:** Detailed information on outcome measures that will be obtained from the CABC^a^ survey and the CRN PROMs^b^ survey.

Outcome(s)				Assessed with		Number of items, n
**CABC survey: ≥7, 13, 24, and 35 months after diagnosis**						
	**Primary outcome (from 7 to 13 months after diagnosis)**					
		Perceived stress	Cohen 10-item Perceived Stress Scale [76]		10	
	**Secondary outcomes (all time points)**					
		Perceived stress	Cohen 10-item Perceived Stress Scale [76]		10	
		Health and health-related quality of life	RAND Corporation 36-Item Short Form Health Survey [11]		36	
		Coping	Theoretically Originated Measure of the Cognitive Activation Theory of Stress [77]		7	
		Fatigue^c^	Chalder’s Fatigue Scale [78]		11+2^d^	
		Awareness/mindfulness	Baer’s Mindfulness Questionnaire [49,79]		15	
		Distress	Patient Health Questionnaire [80]		4	
		Sleep	The Survey of Shift Work, Sleep and Health [81]		10	
		Work and use of social services^e^	Work, use of social services, and Ability Index [82] and single-item question [83]		3	
**CRN PROMs survey: ≥21 days and 14 and 36 months after diagnosis**						
	**Secondary outcomes (all time points)**					
		Cancer-related quality of life	European Organization of Research and Treatment of Cancer-Core Quality of Life Questionnaire [84]		30	
		Breast cancer–related quality of life	European Organization of Research and Treatment of Cancer-Core Quality of Life Questionnaire: Breast Cancer–Specific Module [85]		23	
		Neuropathy/neuropathic pain, vaginal symptoms, arthralgia	Functional Assessment of Cancer Therapy: Gynecologic Oncology Group—Neurotoxicity and Endocrine Symptoms [86]		8	
		Fatigue	Chalder’s Fatigue Scale [78]		11+2^d^	
		Workability and use of social services	Work Ability Index [82] and single-item question [83]		3	
		Stress^f^	Stress Experience Scale [87]		1	
		Background questions	Educational level, living with children <18 years (yes/no), household income, living situation, income, physical activity level (light and high intensity), smoking and alcohol habits, and height and weight		11	

^a^CABC: Coping After Breast Cancer.

^b^CRN PROMs survey: Cancer Registry of Norway patient-reported outcome measures survey.

^c^Chalder’s Fatigue Scale is only included at baseline and at month 24 by the CABC survey because it is assessed with the CRN PROMs survey at 13 and 36 months after diagnosis.

^d^The survey sent to participants diagnosed in 2020 included the 11-item Chalder’s Fatigue Scale, whereas questions on duration and extent of fatigue symptoms [88,89] were added to the survey sent to participants enrolled and followed up after 2021.

^e^Only included at 24 months of follow-up in the CABC survey.

^f^Stress assessed as 1 item is not defined as a secondary outcome but can be used in exploratory analysis.

#### The CRN PROMs Survey

The CRN PROMs survey is sent to all patients with breast cancer 21 days and 14 and 36 months following diagnosis. Several measures from the CRN PROMs survey are included as secondary outcomes in this study as outlined in Table 1.

Fatigue is assessed using variants of Chalder’s Fatigue Scale rating subjective physical and mental features of fatigue [78,88,89]. Peripheral neuropathy, vaginal symptoms, and arthralgia are measured by the Functional Assessment of Cancer Therapy/Gynecologic Oncology Group—Neurotoxicity and Endocrine Symptoms questionnaire [86] (only available for patients diagnosed from 2021). Quality of life is assessed by the 30-item European Organization of Research and Treatment of Cancer-Core Quality of Life Questionnaire (EORTC QLQ-C30) [84] and its Breast Cancer–Specific Module (EORTC QLQ-BR23) [85]. Report on work-related outcomes including use of social services is assessed with the Work Ability Index [82] and single-item question [83].

The CRN PROMs survey also contains self-reported information on background data including educational level, living situation, number of children less than 18 years, income, physical activity level, smoking and alcohol habits, height, and weight (Table 1).

#### Coping After Breast Cancer Questionnaire

Participants receive the baseline CABC survey at approximately 7 months after diagnosis and the follow-up CABC surveys 13, 24, and 35 months after diagnosis (Figure 1). The follow-up surveys were sent about 1 month after the CRN PROMs surveys, in order for participants not to confuse the 2 data collections.

The Cohen 10-item Perceived Stress Scale [76] in the CABC survey will be used to assess the primary outcome: 6-month change in perceived stress (from 7 to 13 months following diagnosis) between each of the intervention groups and TAU (Table 1).

The following secondary outcomes are also obtained from the CABC survey: distress ranging from sadness and worry to anxiety and depression, as measured by the Brief Version of the Patient Health Questionnaire for Depression and Anxiety [80]. (Health-related) quality of life is measured by the RAND Corporation 36-Item Short Form Health Survey (RAND-36). RAND-36 assesses 8 health concepts: physical functioning, role limitations caused by physical health problems, role limitations caused by emotional problems, social functioning, emotional well-being, energy/fatigue, pain, and general health perceptions [11]. Mindfulness is assessed using the 15 items from Baer’s 5 Facet Mindfulness Questionnaire [49]. Sleep (duration and quality) is measured by items from the Norwegian Shift Work, Sleep and Health survey [81]. Coping, defined as 3 dimensions of response outcome expectancies related to an individual’s ability to handle stressors and challenges in everyday life related to coping, hopelessness, and helplessness, is measured by the Theoretically Originated Measure of the Cognitive Activation Theory of Stress [77].

As there is no CRN PROMs survey 24 months after diagnosis, the work-related outcomes are included in the CABC survey after 24 months, whereas Chalder’s Fatigue Scale is included at baseline and at month 24 (Table 1).

#### Other Outcomes

The study will further explore characteristics of those who respond to the CABC trial (responders versus nonresponders) and those who complete the interventions (at least 7 out of 10 modules of StressProffen-CBI or StressProffen-MBI) and their counterparts. Data from the CRN PROMs survey 21 days prior to diagnosis will be used to assess differences in CABC trial invitation responders and nonresponders. Differences in intervention effects (primary and secondary outcomes) between StressProffen-CBI and StressProffen-MBI will also be explored.

Participant data will be linked to the Norwegian Labor and Welfare Administration database, Statistics Norway, the CRN, the Norwegian Patient Registry, the Norwegian Prescription database, and the Norwegian Cause of Death Registry. In addition to data from the surveys, data from the registries will be used for exploratory analysis, including predictors of intervention effects and long-term effects of study participation on comorbidities, relapse and new cancers, and mortality. Detailed and objective information on work participation including sick leave, disability pension, and use of social services will also be obtained. These linkages will enable long-term follow-up of participants in the CABC trial until study end (currently 2035).

### Monitoring Compliance

Those who have not completed the CRN PROMs survey and the CABC survey automatically receive 1-2 reminders (unless they have opted out of receiving reminders) during a 30-day period. To ensure as high participation rate as possible, the CABC survey sent 13 months after diagnosis is available for 45 days and includes 2 additional reminders: 1 by phone and 1 by a standard motivational letter. There is no manual review or monitoring of survey responses during the trial. Survey data and participant data (ie, name, phone number, randomization string, scheduled and actual phone calls, and participant dropouts) are stored in CRN databases protected with 2-factor authentication. Access to survey data is limited to selected employees from the CRN, while participant data are further restricted to employees involved in the CABC study.

In addition to using survey data for monitoring compliance, system usage data (eg, the number of modules completed and time spent on each module and between modules) are logged and stored at the Services for Sensitive Data at the University of Oslo. Only approved study personnel have access to this information prior to study end.

### Pilot Study

Before study implementation in 2021, a pilot study with 23 women (of 59 invited) was conducted to inform the need for adjustments prior to the RCT. Adjustments included minor rewordings of the informed consent, scheduling additional follow-up calls, and reducing the number of items in the CABC survey (eg, the 24-item version of Baer’s Mindfulness Questionnaire [90] was replaced with the 15-item version [49,79]).

### Ethical Considerations

Data collection in the CRN PROMs survey is based on informed consent—Article 6 (1) (a) and Article 9 (2) (a) of the European Union’s General Data Protection Regulation. The Regulations on Population-Based Health Surveys give additional conditions for data collection and processing and what data can be accessed in this study.

All participants are asked to provide informed consent by responding to the CABC survey. Participants are informed that consent also implies consent to have their data linked to several national registries. This includes the CRN; the Norwegian Breast Cancer Registry, where cancer subtypes are recorded; the Norwegian Patient Registry; the Norwegian Cause of Death Registry; and the Norwegian Prescription Database (for comorbidity, morbidity, mortality, and prescription data). Data are also linked with the Norwegian Labor and Welfare Administration Database and Statistics Norway to obtain information on work participation including sick leave and disability. Registry linkages can currently be performed until the end of the study (ie, up to December 31, 2035).

As the intervention is digital, including digitally completed outcome measures, no methods to identify adverse effects have been identified. Further, there is no monitoring committee and hence no stopping guidelines have been implemented. Participants can withdraw from the study at any time and without stating their reasons for doing so. This will not have any consequences for their further treatment. Participants are also free to stop using the app whenever they wish to do so.

The study is approved by Regional Committee for Medical Research Ethics Southeast Norway (reference number 32637 “Stressmestring etter brystkreft” [CABC] and registered at ClinicalTrials.gov, NCT04480203 [CABC]).

### Sample Size Calculations

Power calculation in the CABC trial was conducted on the primary outcome (ie, 6-month between-group difference in perceived stress) using independent sample *t* tests. The α level was adjusted for multiple comparisons (comparing 2 intervention groups with 1 control group for each outcome) by setting the significance level to 2.5%. Estimates of effect sizes and SDs were retrieved from previous StressProffen interventions on multiple groups of patients with cancer, which found a mean difference in perceived stress of 2.8 and SDs of 6.6 and 8.3 in the case and control groups, respectively [70]. As the CABC trial includes a more homogenous population (only female patients with breast cancer), the effect size was set to 3 and the SD to 6.

With an estimated 80% power to detect a difference between 1 of the intervention groups and the control group, a sample size of 235 participants is needed. With an assumed completion rate of 70%, a total of 335 participants will need to be enrolled, 70 in each group.

### Statistical Analysis

Between-group differences in primary and secondary outcomes will be analyzed using intention-to-treat and per-protocol analyses. Continuous outcomes will be analyzed using standard *t* tests (2-tailed and unpaired) and multivariable linear and mixed regression models to adjust for potential differences between groups occurring by chance. Categorical outcomes will be analyzed with chi-square tests and multivariate logistic regression analyses. When analyzing longitudinal data, multivariate regressions with clustered standard errors, multilevel models, or generalized estimating equations may be used to account for the correlation between repeated measurements.

Potential interaction effects between the interventions and relevant covariates such as age at diagnosis, disease characteristics (triple negative, HER2+, or DCIS), and primary treatment will also be explored, either by stratification or by estimating multivariate models including interaction effects.

Missing data will be handled using multiple imputation techniques, primarily if the proportion of participants with missing information exceeds 15%. A detailed description of statistical analysis will be developed. All statistical analyses will be performed using STATA statistical software version 15 or later (StataCorp LLC).

## Results

During inclusion there were signs that the completion rate among participants was lower than expected. By contrast, during the pandemic, a higher than expected percentage of newly diagnosed patients with breast cancer were digitally active (88% in 2022), which resulted in an increase in the percentage of eligible patients with breast cancer that were invited to the CRN PROMs survey in 2022. This provided an opportunity to account for the lower completion rate by recruiting more participants. The target number was therefore increased from 335 to 430 participants (about 100 in each group; enrollment end date May 31, 2023). As of April 14, the total number recruited is 428.

The project has received funding from the Norwegian Breast Cancer Society’s “Pink Ribbon” charity funding (principal investigator: GU).

## Discussion

### Impact of Stress Management

Psychosocial and physical long-term complaints after breast cancer treatment remain a major public health challenge with substantial social and personal costs [9]. Psychosocial stress can be associated with inflammation, tumor progression, and disease prognosis [55,91,92], as well as increased risk and prognosis of other chronic diseases [93,94]. Stress management interventions have been effective in reducing stress and other long-term complaints following a cancer diagnosis [64]. Cancer survivors using the original StressProffen intervention (ie, with a combination of CBT and MBI) over a 12-month study period reported significantly greater reduction in perceived stress, depression, and anxiety compared with controls (ie, TAU). The intervention also showed significantly improved health-related quality of life for cancer survivors receiving StressProffen compared with controls [71].

The CABC trial will study similar outcomes as in the original StressProffen intervention, but solely in breast cancer survivors and with additional long-term follow-up. If any or both of the current interventions have effects that are superior to findings from the TAU control group, implementation of the convenient eHealth intervention StressProffen should be considered in clinical practice, perhaps as a future extension to precision oncology care [55].

The original StressProffen program (ie, the app) targeting cancer survivors is in the process of being made commercially available in English and Norwegian versions, whereas the StressProffen-CBI and MBI versions are only available for study participants. Future distribution will depend on study findings.

### Strengths

The CABC trial is, to our knowledge, currently the largest psychosocial eHealth intervention (RCT) in patients with breast cancer. The trial has a long-term follow-up with an assessment of intervention effects 1, 2, and 3 years after a breast cancer diagnosis, corresponding to up to 29 months after baseline. Furthermore, study participants can be prospectively followed up with respect to comorbidity, relapse and new cancers, and mortality until the end of the study (ie, currently, December 31, 2035) through linkage with national health registries using personal identification numbers (mandatory for all Norwegians). The feasibility of the StressProffen app was assessed initially by Børøsund and colleagues [71] and the CABC trial has further extended the StressProffen research line through developing the StressProffen-CBI and StressProffen-MBI interventions, pilot tested these, and implemented necessary adjustments. Another strength of the trial is the ongoing systematic collection of CRN PROMs survey data. This enables us to compare study participants with all CRN PROMs survey data.

Development of the 2 interventions strived to balance design and content presentation to enhance the chance of any effect differences arising due to theoretical content (ie, CBI/MBI). However, due to statistical power limitations, direct comparisons of the 2 interventions will solely be conducted on an exploratory basis. The flexibility of the intervention can be beneficial for patients with cancer undergoing treatment while coping with everyday activities [64]. Given the high percentage of the population that has access to digital platforms within Norway [68], the study has a unique possibility to recruit participants across different socioeconomic statuses and geographical locations. Digital intervention delivery also demands less resources than face-to-face interventions [56]. Thus, if found to be effective, the StressProffen eHealth intervention may have a better chance of being integrated as part of clinical practice than costly face-to-face interventions.

### Limitations

The CABC trial has some limitations. First, outcome measures are primarily self-reported. Second, data related to outcomes prior to breast cancer diagnosis are not available. Third, the study only includes a subsample of patients with breast cancer with either noninvasive disease (DCIS) or severe (triple-negative or HER2 positive) breast cancer. Participants are therefore likely to be a heterogenous group of patients with breast cancer with varying treatment according to stage and subtype (eg, in addition to surgery, patients with DCIS may only receive radiation therapy, whereas patients with triple-negative or HER2-positive cancer are advised chemotherapy for several months) [95]. Selection bias may be present as participation depends on the use or access to the digital mailbox and willingness to participate in the CRN PROMs survey (ie, about 50%) [68]. These factors could also limit the generalizability of the study results. However, an eHealth intervention requires the use of digital resources and access to digital tools as requirements.

### Conclusions

The CABC trial is possibly the largest ongoing psychosocial eHealth RCT in patients with breast cancer. If 1 or both interventions prove to be effective in reducing stress and improving psychosocial and physical complains, the StressProffen eHealth interventions could be beneficial, inexpensive, and easily implementable tools for breast cancer survivors when coping with late effects after cancer and cancer treatments.

## Appendix

Multimedia Appendix 1 Standard Protocol Items: Recommendations for Interventional Trials (SPIRIT) checklist.

## Abbreviations

CABC: Coping After Breast Cancer
CBI: cognitive behavioral intervention
CBT: cognitive behavioral therapy
CRN: Cancer Registry of Norway
DCIS: ductal carcinoma in situ
EORTC QLQ: European Organization of Research and Treatment of Cancer-Core Quality of Life Questionnaire
EORTC QLQ-BR23: European Organization of Research and Treatment of Cancer-Core Quality of Life (Breast Cancer–Specific Module) Questionnaire
EORTC QLQ-C30: European Organization of Research and Treatment of Cancer-Core Quality of Life Questionnaire
ER: estrogen receptor
HER2: human epidermal growth factor receptor 2
ICD-10: International Classification of Diseases, 10th revision
MBI: mindfulness-based intervention
PROM: patient-reported outcome measure
RAND-36: RAND Corporation 36-Item Short Form Health Survey
RCT: randomized controlled trial
SPIRIT: Standard Protocol Items: Recommendations for Interventional Trials
TAU: treatment as usual

## Availability of Data and Materials

The informed consent as well as the General Data Protection Regulation will limit the extent to which individual-level data can be shared with other researchers.
